# ConvNet-Generated Adversarial Perturbations for Evaluating 3D Object Detection Robustness

**DOI:** 10.3390/s25196026

**Published:** 2025-10-01

**Authors:** Temesgen Mikael Abraha, John Brandon Graham-Knight, Patricia Lasserre, Homayoun Najjaran, Yves Lucet

**Affiliations:** 1 Computer Science, University of British Columbia Okanagan, Kelowna, BC V1V 1V7, Canada; temex@student.ubc.ca (T.M.A.); bgk60@student.ubc.ca (J.B.G.-K.); patricia.lasserre@ubc.ca (P.L.); 2 Faculty of Engineering and Computer Science, University of Victoria, Victoria, BC V8P 5C2, Canada; najjaran@uvic.ca

**Keywords:** robust artificial intelligence, sensitivity analysis, point clouds, object detection, computer vision, deep learning, adversarial attacks, autonomous driving, 3D perception, LiDAR

## Abstract

**Highlights:**

**What are the main findings?**

- Our adversarial Convolutional Neural Network (ConvNet) generates imperceptible perturbations that degrade 3D object detection by 8–24% across KITTI and NuScenes datasets.
- Smaller objects (pedestrians, cyclists) show 3x higher vulnerability to adversarial attacks compared to larger vehicles.

**What is the implication of the main finding?**

- Current state-of-the-art 3D detection systems used in autonomous vehicles are vulnerable to subtle adversarial perturbations within sensor noise margins.
- Safety-critical applications require robust defense mechanisms, especially for protecting vulnerable road users where detection failures pose the greatest risk.

**Abstract:**

This paper presents a novel adversarial Convolutional Neural Network (ConvNet) method for generating adversarial perturbations in 3D point clouds, enabling gradient-free robustness evaluation of object detection systems at inference time. Unlike existing iterative gradient methods, our approach embeds the ConvNet directly into the detection pipeline at the voxel feature level. The ConvNet is trained to maximize detection loss while maintaining perturbations within sensor error bounds through multi-component loss constraints (intensity, bias, and imbalance terms). Evaluation on a Sparsely Embedded Convolutional Detection (SECOND) detector with the KITTI dataset shows 8% overall mean Average Precision (mAP) degradation, while CenterPoint on NuScenes exhibits 24% weighted mAP reduction across 10 object classes. Analysis reveals an inverse relationship between object size and adversarial vulnerability: smaller objects (pedestrians: 13%, cyclists: 14%) show higher vulnerability compared to larger vehicles (cars: 0.2%) on KITTI, with similar patterns on NuScenes, where barriers (68%) and pedestrians (32%) are most affected. Despite perturbations remaining within typical sensor error margins (mean L2 norm of 0.09% for KITTI, 0.05% for NuScenes, corresponding to 0.9–2.6 cm at typical urban distances), substantial detection failures occur. The key novelty is training a ConvNet to learn effective adversarial perturbations during a one-time training phase and then using the trained network for gradient-free robustness evaluation during inference, requiring only a forward pass through the ConvNet (1.2–2.0 ms overhead) instead of iterative gradient computation, making continuous vulnerability monitoring practical for autonomous driving safety assessment.

## 1. Introduction

There is an increase in the popularity of 3D point clouds for various applications, including self-driving cars, model reconstruction, drone applications, and quality inspection in construction and manufacturing. In most of these applications, trained deep neural networks are employed for 3D object classification and segmentation. Some of these applications have privacy, safety, or security concerns that must be satisfied. Face recognition, autonomous driving, voice command recognition, and fraud detection are examples of security-critical applications. Recent works have demonstrated that network inputs can be intentionally manipulated to create incorrect predictions [1,2]. More concerning, such incorrect predictions often have high certainty levels. In computer vision tasks in particular, small perturbations can result in shifts in predictions even for state-of-the-art algorithms [3]. These perturbations are often imperceptible to humans.

Deep neural networks have been proven vulnerable to adversarial attacks carefully designed to fool the system. An early exploration of adversarial attacks raised questions about the ability of neural networks to generalize. Szegedy et al. [4] and Goodfellow et al. [3] demonstrated that virtually imperceptible changes to the input image can lead to incorrect predictions in contemporary models. The Jacobian-based Saliency Map Attack (JSMA) [5] shows that large output variations can be achieved even when the perturbations are applied to a small portion of input features.

To ensure that safety-critical applications utilize a highly efficient algorithm to detect objects accurately, it is crucial to understand how adversarial attacks can impact the neural networks employed in such algorithms. The understanding of adversarial attacks can also provide direction in designing defensive systems [6,7,8]. Although there has been rigorous research on 2D point clouds, the interest in 3D point clouds is relatively recent [9,10,11,12]. In this paper, we propose and evaluate a novel method of generating and injecting adversarial perturbations to 3D point clouds. The method is applied to a pretrained model used for car detection in the KITTI dataset, taken from the mmdetection3d model zoo.

**Figure 1:**
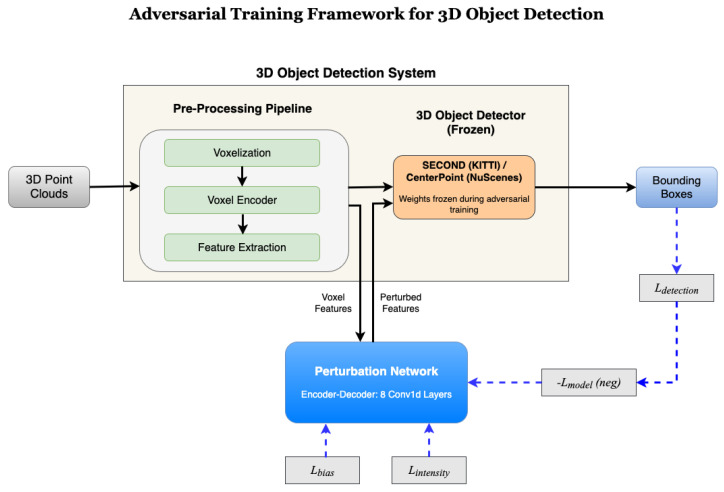
Adversarial training framework for 3D object detection. The system processes 3D point clouds through a preprocessing pipeline (voxelization, voxel encoding, and feature extraction) before entering the detection network. Our Perturbation Network generates adversarial perturbations from voxel features, which are combined with original features to create perturbed inputs for the frozen 3D object detector (SECOND for KITTI, CenterPoint for NuScenes). The detailed architecture of the Perturbation Network’s encoder–decoder structure is provided later in the manuscript. During training, the Perturbation Network learns through backpropagation (dashed blue arrows) from multiple loss components: $-L_{\mathrm{model}}$ (negative detection loss for adversarial objective), $L_{\mathrm{bias}}$ (preventing directional shifts), and $L_{\mathrm{intensity}}$ (constraining perturbation magnitude). The detector weights remain frozen during adversarial training to focus optimization on generating effective perturbations.

Our approach differentiates itself by employing an adversarial Convolutional Neural Network (ConvNet) to introduce perturbations into the 3D point cloud; this ConvNet can be used gradient-free during inference to approximate robustness. Training the ConvNet is undertaken using a whitebox method. Figure 1 shows the outline of our proposed method. We balance the perturbations across both point cloud dimension and perturbation direction by introducing these terms to the training loss function. We evaluate the method using Average Precision (AP) and Intersection-over-Union (IoU) for four standard metrics and stratify results over easy, medium, and hard examples.

**Figure 2:**
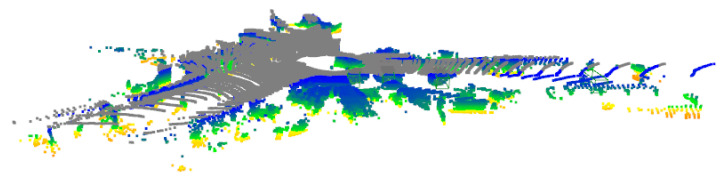
Side-view perspective of a KITTI dataset scene showing 3D point cloud data captured by vehicle-mounted LiDAR. Colors represent height: yellow (elevated structures), blue-green (mid-level objects), and gray (ground plane). The scene extends approximately 70 m forward from the sensor position, capturing various vehicles and road infrastructure with decreasing point density at greater distances.

Figure 2 shows a typical 3D driving scene from the KITTI dataset where our method would be applied. The point cloud representation captures objects at varying distances with different densities, which is the type of data our adversarial ConvNet processes after voxelization.

In summary, our contributions in this paper are as follows:

1. We present a novel way to evaluate robustness gradient-free at inference, using a ConvNet to generate targeted adversarial perturbations. While there has been some previous research on adversarial attacks on 3D point cloud detection systems, to the best of our knowledge, none of these approaches have utilized the structure of the detection system to their advantage. We propose a model in which the adversary leverages knowledge about the detection system to design targeted attacks.
2. We demonstrate the effectiveness of our attacks by testing them on two state-of-the-art detection systems, Sparsely Embedded Convolutional Detection (SECOND) on KITTI and CenterPoint on NuScenes, showing that detection efficiency significantly falls across different architectures and datasets.
3. We evaluate impact using mean Average Precision (mAP) across difficulty levels for KITTI and both mAP and NuScenes Detection Score (NDS) for NuScenes. We identify a clear inverse correlation between object size and adversarial vulnerability, with smaller objects (such as pedestrians and cyclists) showing a disproportionate susceptibility to attacks.

The primary objective of this research is to develop a gradient-free method for evaluating the robustness of 3D object detection systems against adversarial perturbations across all object classes. While our method is designed for robustness evaluation, our findings reveal that smaller objects (pedestrians, cyclists) exhibit higher vulnerability, which has critical implications for autonomous vehicle safety. The rest of this paper is organized as follows: Section 2 reviews related work, Section 3 provides theoretical foundations, Section 4 presents our methodology, Section 5 evaluates our approach, Section 6 discusses implications, and Section 7 concludes this paper.

## 2. Related Work

Adversarial attacks can be classified based on the frequency of the attack, falsification method (increasing false positives or false negatives), prior knowledge of the network architecture and parameters, and whether the disturbance is applied to change the network output to a specific or random prediction. Comprehensive categorizations are provided in [1,2].

Deepfool defines the robustness of a binary classifier as the distance to the decision boundary. For affine binary classifiers, this boundary is a hyperplane, and a closed-form solution exists to find the minimum disturbance required for a prediction to cross this hyperplane. This approach is extended to multi-class and non-linear functions, with the disturbance calculated using an iterative approach on a linear approximation of the function around the test point [13].

Early work on applying deep learning to point clouds resulted in VoxelNet, which replaced hand-crafted feature representations with deep learning-based feature extraction [14]. Similarly, PointNet++ pioneered deep learning on non-uniformly sampled point clouds, applied to both segmentation and classification tasks [15]. PointPillars extended PointNet with a 2D CNN (Convolutional Neural Network) backbone and a detection head, achieving state-of-the-art results on the KITTI dataset [16]. SECOND provided faster training and inference for deep learning methods on point clouds through sparse convolution; SECOND also introduced directional loss and improved data augmentation techniques [17]. CenterPoint [18] extends center-based 2D detection methods to 3D, achieving state-of-the-art performance on NuScenes through center heatmap prediction and refined 3D box regression.

Recent advancements in 3D adversarial attacks have led to innovative methods tailored to different representations of 3D data, achieving a balance between adversarial strength and imperceptibility. Lou et al. [19] introduced HiT-ADV, an imperceptible shape-based adversarial method that targets complex surface areas of 3D objects. This technique utilizes a two-stage search based on saliency and imperceptibility scores, employing deformation perturbations with Gaussian kernel functions to maintain subtlety while enhancing adversarial strength. HiT-ADV extends to physical attacks through benign resampling and rigid transformations, demonstrating superior effectiveness in both digital and physical realms.

Similarly, Huang et al. [20] developed a novel approach for generating shape-invariant adversarial perturbations on 3D point clouds. Their method, based on a Point-Cloud Sensitivity Map, applies reversible coordinate transformations to constrain movement to tangent planes and uses gradient-based calculations to determine optimal attack directions. This approach preserves the shape surface while guiding perturbation generation, showing significant improvements in attack efficiency and imperceptibility across various point cloud recognition models.

Furthermore, Zhang et al. [21] proposed the Mesh Attack method, which perturbs the mesh representation of 3D objects. This method incorporates a differential sampling module that facilitates gradient backpropagation and employs chamfer, Laplacian, and edge length losses to ensure the adversarial meshes are smooth and 3D printable. Demonstrated through extensive testing, Mesh Attack outperforms existing 3D adversarial techniques in robustness and performance under various defensive scenarios.

In addition to these methods, Zhang et al. [22] conducted a comprehensive study on the robustness of Light Detection and Ranging (LiDAR)-based 3D detectors against adversarial attacks, introducing three distinct attack types: point perturbation, point detachment, and point attachment. They extended these attacks to the 3D object detection task and benchmarked their effectiveness against state-of-the-art LiDAR-based 3D object detectors on large-scale datasets such as KITTI and Waymo. Their results highlighted the varying degrees of robustness in voxel-based methods when subjected to different types of attacks, emphasizing the need for more robust detection systems in safety-critical applications.

Building on these foundational attack strategies, recent work has explored various approaches to improve adversarial attacks on 3D perception systems. Mahima et al. [23] systematically evaluated the adversarial robustness of LiDAR semantic segmentation networks, demonstrating vulnerabilities to point perturbation, injection, and removal attacks, with the Cylinder3D network showing the highest susceptibility. Cai et al. [24] proposed the Contextual Attribution Maps-Guided Attack (CAMGA) for 3D object detection, which targets contextual regions rather than objects directly. This approach achieves attack success rates exceeding 68% on large-scale datasets by exploiting models’ over-reliance on contextual information. While these methods demonstrate effective attack strategies for 3D perception tasks, they primarily focus on traditional perturbation methods, highlighting the gap addressed by our ConvNet-based approach that enables gradient-free robustness evaluation at inference time.

Combining multiple predictors is a viable path to improving deep learning-based point cloud methods. MVX-Net fused both point cloud and camera data for 3D object detection. VoteNet, on the other hand, combined predictions generated from only point clouds without relying on RGB images; predictions were combined using Hough Voting [25]. Imvotenet combined these two ideas, using information from 2D RGB images to narrow the 3D search space from which the predictors were generated [26].

Efficiency is also a concern in processing 3D point clouds, which is particularly essential in edge applications with limited or no connectivity to cloud-based computational power. 3DSSD removed feature propagation layers and prediction refinement; instead, they introduced a novel sampling strategy which retained positive points for localization and negative points for classification [27]. PointRCNN simplifies the detection of complex objects by first detecting intra-object parts; these parts are aggregated to score bounding boxes and refine localization. However, this method relies on domain knowledge regarding the composition of objects [28].

Due to strong commercial interest in self-driving cars, common datasets utilizing 3D point clouds include street scenes with the intention of facilitating autonomous navigation. Examples of such datasets include KITTI [29], Waymo [30,31], NuScenes [32], and Lyft [33]. Recent work has also highlighted the broader implications of adversarial robustness in transportation systems. Zhai et al. [34] demonstrated how cyber-attacks can affect connected automated vehicles through lattice hydrodynamic modeling, showing that adversarial perturbations can disrupt the stability of traffic flow. Similarly, Liu et al. [35] developed intelligent detection systems for foreign object intrusion in subway stations, highlighting the importance of robust detection in safety-critical transportation infrastructure.

While these existing approaches have advanced adversarial attacks on 3D point clouds, they typically operate as external perturbation methods without leveraging the internal structure of detection pipelines. This gap motivates our approach of embedding an adversarial ConvNet directly within the detection architecture, enabling gradient-free robustness evaluation at inference time while maintaining perturbations within realistic sensor error bounds. By integrating the attack mechanism into the voxel feature processing stage, we can generate more targeted and effective perturbations that exploit the specific vulnerabilities of modern 3D object detectors.

Table 1 summarizes the key differences between our approach and existing 3D adversarial attack methods. Our ConvNet-based approach offers several unique advantages: (1) it is the only method enabling gradient-free robustness evaluation at inference time through a learned adversarial network, (2) it integrates directly into the detection pipeline at the voxel feature level rather than manipulating raw points, and (3) it has been validated across multiple architectures (SECOND and CenterPoint) and datasets (KITTI and NuScenes). Note that Zhang et al. [22] provides a comprehensive benchmark study evaluating multiple attack types rather than proposing a single new method. The gradient-free nature of our method enables efficient robustness testing at inference time, making it practical for continuous monitoring in safety-critical applications. While other methods require iterative gradient computation during inference, our approach uses a single forward pass through the trained ConvNet, thereby improving evaluation efficiency.

## 3. Background

The sensitivity of a model is generally defined as the change in output relative to the change in input; a model that generates large changes in output for small changes to input is said to be highly sensitive. Mathematically, this concept is captured in the Lipschitz constant. Given a function $f:R^{n}\to R^{m}$, *f* is Lipschitz continuous if its maximum rate of change is bounded by some non-negative constant; specifically, on the set $\chi \subseteq R^{n}$, the function must satisfy

(1) ∥f(x)−f(y)∥≤L∥x−y∥,∀x,y∈χ,

where *L* is a non-negative constant and the smallest possible *L* is called the Lipschitz constant; such a function *f* can be called *L*-Lipschitz, denoted as L(f). Intuitively, the Lipschitz constant gives the maximum change in the output given a fixed change in the input.

The Lipschitz constant can be seen as a measure of the robustness of the function *f*; when the Lipschitz constant is small, large changes in the output are possible only given large changes in the input. For simple, known functions, this bound can be calculated analytically. For more complex functions, such as neural networks, it is common to seek an approximation or upper bound of *L* [7,36].

A feed-forward neural network can be expressed as successive applications of functions, where each function is a layer of the network:

(2) $$\hat{Y}=F\left(X\right)=\left(f_{l}◦f_{l-1}◦\ldots ◦f_{1}\right)\left(X\right).$$

From [7], a worst-case upper bound on *L* can be found through the product of the Lipschitz constants of the layers:

(3) $$L\left(X\right)\leq \prod_{i=1}^{l}L\left(f\left(i\right)\right).$$

Notably, this worst-case upper bound can be very pessimistic, even if the Lipschitz constants of the underlying layers are the best possible. Still, an understanding of the Lipschitz constants of the underlying layers can be useful in informing the sensitivity of the network. For fully connected layers of the form

(4) f(X)=WX+b

the Lipschitz constant can be derived from (1), yielding the operator norm of *W*:

(5) $$L\left(f\right)=\underset{X_{1}-X_{2}\neq 0}{\sup}\frac{∥W\left(X_{1}-X_{2}\right){∥}_{p}}{∥X_{1}-X_{2}{∥}_{p}}.$$

Common norm values *p* include the 1-norm (sum of parameter absolute values), 2-norm (Euclidean distance), and *∞* (maximum value); while technically not satisfying the properties of a norm, a value of 0 is also commonly used in machine learning to denote the count of non-zero parameters. The authors continue with a similar analysis for convolutional layers. They further show that *L* for pooling, ReLU activation, and softmax layers is 1.

Pointwise robustness is defined as the smallest perturbation for which test examples within the perturbation distance still result in the correct label. Adversarial frequency measures the number of adversarial examples within a threshold distance to a test sample that have a different label than the test sample. Adversarial severity measures the severity with which a network is not pointwise robust at a given point in the input space. Severity is measured as a distance threshold to the test sample; smaller distance thresholds translate to worse severity, as smaller perturbations generate false predictions [37].

## 4. Materials and Methods

### 4.1. Adversarial Perturbation Method

This paper introduces adversarial perturbations into a neural network used for object detection in 3D point clouds. The method employs an adversarial ConvNet to generate targeted perturbations that are integrated directly into the detection pipeline. The ConvNet seeks to maximize prediction loss while maintaining subtle, imperceptible perturbations through carefully designed loss constraints.

Our method is designed for deployment in autonomous vehicle perception systems where 3D point clouds must be processed in real time. The approach enables robustness evaluation during both development testing and operational deployment, providing vulnerability metrics without requiring gradient computation at inference time. This is particularly relevant for robustness testing and continuous monitoring of perception systems, where the 1.2–2.0 ms inference overhead allows practical integration into existing pipelines. The method operates on voxelized representations of 3D scenes, where objects at different distances and of varying sizes must be reliably detected for safe navigation.

#### 4.1.1. Architecture Integration

The pretrained models selected from mmdetection3d consist of the following stages:

1. Voxelization.
2. Voxel Encoder (HardSimpleVFE).
3. Encoder (SparseEncoder for SECOND, modified for CenterPoint).
4. Backbone (SECOND or CenterPoint backbone).
5. Neck (SECONDFPN or CenterPointFPN).
6. Head (Anchor3D for SECOND, CenterHead for CenterPoint).

**Figure 3:**
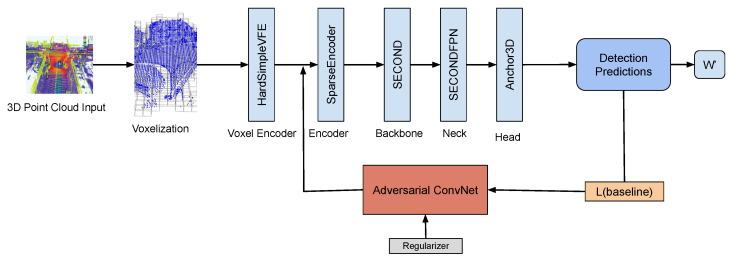
Pretrained model structure and perturbation injection method. The detection pipeline consists of six sequential stages: (1) Voxelization groups point clouds into a 3D grid, (2) HardSimpleVFE (Voxel Encoder) extracts 4-dimensional features (x, y, z, intensity) for KITTI or 5-dimensional features (x, y, z, intensity, timestamp) for NuScenes from each non-empty voxel, (3) our adversarial ConvNet (detailed in Figure 4) injects learned perturbations directly into these voxel features, (4) SparseEncoder [17] applies sparse 3D convolutions to convert the perturbed sparse voxel representation into dense 2D feature maps, (5) the backbone network (SECOND [17] or CenterPoint [18]) processes these features through convolutional layers, and (6) the detection head (Anchor3D for SECOND, CenterHead for CenterPoint) generates final bounding box predictions.

For the whitebox model to be trainable, gradients must be available for backpropagation. Voxelization in the baseline model is a non-differentiable process; therefore, perturbations are applied between steps 2 and 3. There, a ConvNet is introduced, which calculates the perturbation to be applied to each point independently; this perturbation is then summed with the output of step 2 and passed to step 3. This process is displayed in Figure 3.

#### 4.1.2. Adversarial ConvNet Design

**Figure 4:**
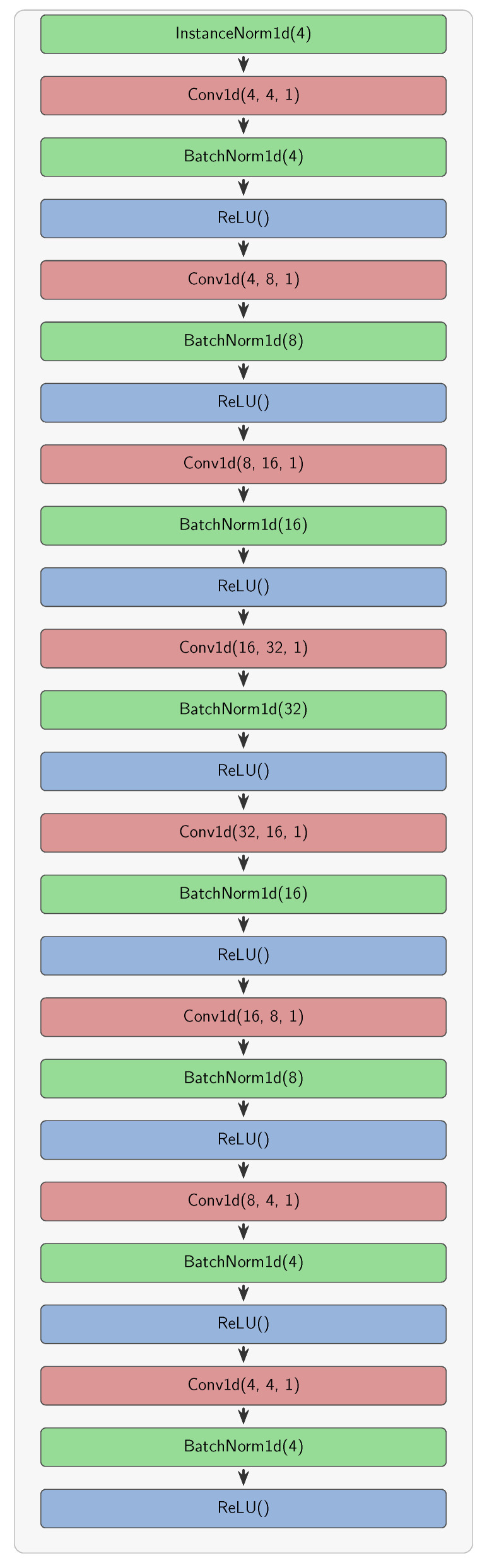
Detailed architecture of the adversarial ConvNet. The network employs an encoder–decoder structure with 8 Conv1d layers, each using kernel size 1 for point-wise operations on individual voxel features. The encoder progressively increases feature dimensions (4→8→16→32) to learn complex perturbation patterns, while the decoder symmetrically reduces dimensions (32→16→8→4) to match the input feature space. Instance normalization preprocesses inputs, with batch normalization and ReLU activations between convolutional layers. This architecture processes variable-length point clouds efficiently while generating constrained perturbations through learned parameters optimized during adversarial training.

The adversarial ConvNet is an overcomplete encoder–decoder architecture with eight convolutional layers, as shown in Figure 4. It automatically adapts to different input dimensions based on the dataset, using four features (x, y, z, intensity) for KITTI and five features (x, y, z, intensity, timestamp) for NuScenes. While the core encoder–decoder structure remains consistent across experiments, training parameters were tuned for each dataset–model combination to account for their different characteristics and robustness levels.

Instance normalization is applied at the start of the network, while batch normalization and the ReLU activation function are applied between convolutional layers. The input to the first convolutional layer corresponds to the number of dimensions in the point cloud readings. The number of features is doubled at each convolutional layer, reaching a maximum of 32 features. Dimensionality reduction is performed in the second half of the network, producing output features that comprise the perturbation. All convolution layers are 1-dimensional with a kernel size of 1, meaning that the network operates on each point in isolation from other points. This also allows the network to accept point clouds of varying lengths. Implemented in PyTorch (v2.0.1+cu118) within the mmdetection3d framework, this design enables efficient gradient-free robustness evaluation at inference time—requiring only a single forward pass through the trained ConvNet (adding just 1.2-2.0 ms latency) rather than iterative gradient computation methods. The adversarial network adds minimal parameters (48,000, less than 1% of the baseline), making it practical for real-time deployment.

#### 4.1.3. Loss Function Design

The loss function is a composition of the baseline loss and measures to control the properties of the perturbations. Baseline loss is the sum of 3 components:

(6) $$L_{baseline}=L_{classification}+0.2\cdot L_{directional}+2\cdot L_{bounding\_box}.$$

The weights (0.2 for directional loss and 2 for bounding box loss) are hyperparameters from the original SECOND implementation [17] that balance the relative importance of classification accuracy, angular precision, and localization accuracy.

The adversarial loss function controls the intensity as well as the bias and imbalance of the perturbations. Intensity loss is calculated as the mean $L_{2}$ norm over all perturbations in a sample:

(7) $$L_{intensity}=\frac{\sum_{p\in P}{∥p∥}^{2}}{\left|P\right|}$$

where *P* is the set of perturbations to be applied, whose length is equal to the number of voxel features. This term serves as the primary constraint on perturbation magnitude, ensuring that modifications remain close to realistic sensor error bounds. The quadratic penalty encourages the majority of perturbations to be small while allowing occasional larger perturbations where necessary for attack effectiveness.

Controlling bias is needed to prevent the model from learning to shift all points in a single direction, moving them outside of the ground-truth annotations. This is calculated as the $L_{2}$ norm of the mean perturbation vector:

(8) $$L_{bias}={∥\bar{p}∥}^{2}\;\mathrm{where}\;\bar{p}=\frac{1}{\left|P\right|}\sum_{p\in P}p$$

where $\bar{p}$ is a vector containing the mean perturbation for each dimension, and each p is a perturbation vector with *D* dimensions. For the KITTI dataset, D=4 (x, y, z, intensity), while for NuScenes, D=5 (x, y, z, intensity, timestamp). This constraint ensures zero-mean perturbations, mimicking natural sensor noise patterns rather than systematic offsets that would be easily detectable through simple statistical analysis.

Controlling imbalance causes the model to prefer small changes in all directions over large changes in a single dimension. This is defined as the standard deviation of the absolute values of the mean perturbations across dimensions:

(9) $$L_{imbalance}=\sigma \left(\{|{\bar{p}}_{1}|,|{\bar{p}}_{2}|,\ldots ,|{\bar{p}}_{D}|\}\right)$$

where σ denotes the standard deviation function applied to the set of scalar values, and ${\bar{p}}_{d}$ represents the *d*-th component of the mean perturbation vector $\bar{p}$. This term prevents the adversarial network from exploiting individual feature channels, ensuring that perturbations affect spatial coordinates and intensity measurements proportionally, thereby maintaining physical plausibility.

These losses are weighted and summed with the negation of baseline loss while training the adversarial network:

(10) $$L=-L_{model}+3L_{intensity}+10L_{bias}+10L_{imbalance}.$$

The weights in this formulation were determined empirically to balance competing objectives. The negative weight on $L_{model}$ creates the adversarial objective, maximizing detection failure through gradient ascent. The weight of 3 for intensity loss maintains perturbations within acceptable bounds while allowing sufficient freedom for effective attacks. The higher weights of 10 for both bias and imbalance losses reflect their critical role in preserving attack realism; violations of these constraints would make perturbations easily detectable through simple filtering or statistical analysis. This multi-objective optimization creates a min-max game where the adversarial ConvNet learns to maximize degradation of detection performance while satisfying physical and statistical constraints. During training, gradient clipping to [−0.1, 0.1] and numerical stability checks ensure convergence despite the competing objectives. The resulting perturbations achieve the delicate balance required for effective yet imperceptible adversarial attacks, as evidenced by our experimental results showing major detection degradation (8–24%) with perturbations averaging less than 0.1% of the point cloud magnitude.

### 4.2. Datasets and Models

#### 4.2.1. KITTI Dataset

The KITTI dataset [29] contains 3712 training and 3769 test samples captured with a Velodyne HDL-64E LiDAR sensor. We evaluate on the 3-class detection task (car, pedestrian, cyclist) using the SECOND detector [17] implemented in mmdetection3d [38]. The dataset provides point clouds with approximately 100,000 points per frame, covering a range of up to 120 m. Results are stratified by difficulty levels (easy, moderate, hard) based on object occlusion, truncation, and bounding box height thresholds defined by the KITTI benchmark.

#### 4.2.2. NuScenes Dataset

The NuScenes dataset [32] provides 28,130 training and 6019 validation samples captured in urban scenarios across Boston and Singapore. We evaluate on the 10-class detection task using CenterPoint [18]. The 10 classes include various vehicle types (car, truck, bus, trailer, construction vehicle, motorcycle, bicycle) and vulnerable road users (pedestrian, barrier, traffic cone). NuScenes uses a 32-beam LiDAR sensor with 20 Hz capture rate, providing denser temporal information but sparser point clouds compared to KITTI. The dataset includes challenging scenarios with heavy occlusion, varied weather conditions, and complex urban environments.

### 4.3. Training and Evaluation Setup

#### Training Configuration

For KITTI experiments with SECOND, optimization used 20 epochs with 3712 training samples. The learning rate was set to 0.001 with a 2x multiplier for adversarial components, and dynamic adversarial scaling up to 4x strength was applied to enhance attack effectiveness. The baseline loss weights represent the KITTI configuration with an adversarial weight of 0.5.

For NuScenes with CenterPoint, we employed 20 epochs with 28,130 training samples. A more conservative learning rate of 5 ×$10^{-5}$ was used with fixed adversarial weights (0.05 versus 0.5 for KITTI) to ensure stable training given CenterPoint’s different architecture and the increased complexity of 10-class detection.

Both configurations utilized the AdamW optimizer [39] with weight decay of 0.01 and a batch size of 6. Training was performed on dual NVIDIA RTX 6000 Ada Generation GPUs with 48 GB memory each, enabling efficient parallel processing of point cloud data. The implementation leverages and modifies the training configurations of the mmdetection3d framework [38].

Furthermore, perturbations are constrained to maintain realism and avoid detection through simple filtering. For KITTI, the maximum L2 perturbation is limited to 0.2 m, accounting for compound error sources including calibration uncertainties, environmental factors, and dynamic scene elements that occur in real-world deployments. In practice, the actual perturbations achieved are substantially smaller than this constraint, averaging only 0.088% of the point cloud magnitude (see Results section for detailed analysis).

### 4.4. Complexity Analysis

The computational overhead of our adversarial method consists of training and inference components. During training, the total complexity is $O(E\cdot N\cdot \left(C_{base}+C_{adv}\right))$, where *E* represents epochs, *N* is the dataset size, $C_{base}$ is the baseline detector complexity, and $C_{adv}$ is the adversarial ConvNet complexity. For KITTI with SECOND, this translates to 20 epochs × 3712 samples, with the adversarial ConvNet adding approximately 48,000 parameters (less than 1% of the baseline’s 5.3M parameters). For NuScenes with CenterPoint, the scale increases to 20 epochs × 28,130 samples, though the adversarial overhead remains proportionally similar.

The adversarial network processes voxel features with complexity $O(P\cdot \sum_{i=1}^{8}w_{i}\cdot c_{i})$ where *P* is the number of non-empty voxels (typically 15,000–20,000 for KITTI and 10,000–15,000 for NuScenes), and $w_{i}$ and $c_{i}$ represent the input and output channels at each Conv1d layer. With eight convolutional layers using kernel size 1, the architecture progresses through channels [4→4, 4→8, 8→16, 16→32, 32→16, 16→8, 8→4, 4→4], resulting in (4×4+4×8+8×16+16×32+32×16+16×8+8×4+4×4)=1376 operations per point, or approximately O(20K·1.4K)≈28M operations per forward pass.

At inference, the adversarial ConvNet requires a single forward pass, adding O(P·L) operations where L=8 Conv1d layers, resulting in approximately 1.2–2.0 ms additional latency on an NVIDIA RTX 6000 Ada Generation (3–5% overhead on the baseline’s 40 ms). Memory requirements increase by approximately 120 MB during training to store intermediate activations and adversary gradients for the min-max optimization, while inference memory overhead is negligible (approximately 200 KB for the ConvNet parameters in FP32).

The training process incorporates several computational safeguards: gradient clipping to [−0.1, 0.1] prevents explosion during adversarial training, batch normalization with momentum 0.1 stabilizes the optimization, and the NaN detection hook (checking every iteration) adds minimal overhead (<0.1%) while ensuring numerical stability. The min-max optimization alternates between maximizing detection loss for the adversary and minimizing it for the detector, requiring careful learning rate scheduling (0.001 for KITTI, 5 × 10^−5^ for NuScenes) to achieve convergence.

### 4.5. Evaluation Metrics

#### 4.5.1. KITTI Metrics

For KITTI evaluation, we employ AP calculated at different IoU thresholds following the official KITTI benchmark protocol. The IoU threshold is set to 0.7 for cars and 0.5 for pedestrians and cyclists, reflecting the different localization requirements for various object sizes. Performance is reported across three difficulty levels:

- Easy: Fully visible objects with height >40 pixels, occlusion level =0.
- Moderate: Partly occluded objects with height >25 pixels, occlusion level ≤1.
- Hard: Difficult-to-see objects with height >25 pixels, occlusion level ≤2.

#### 4.5.2. NuScenes Metrics

For NuScenes, we report two primary metrics:

- mAP: Calculated as the average precision across all 10 classes at various distance thresholds (0.5 m, 1 m, 2 m, 4 m) and averaged over matching thresholds.
- NDS: A composite metric that incorporates mAP along with translation, scale, orientation, velocity, and attribute errors. NDS provides a more comprehensive evaluation of detection quality beyond simple localization accuracy.

Both metrics are computed using the official NuScenes evaluation toolkit to ensure reproducibility and comparability with other methods. The weighted average accounts for the frequency of each class in the dataset, providing a balanced assessment of overall system performance.

## 5. Results

### 5.1. KITTI Dataset Results

Our enhanced adversarial training on the KITTI three-class dataset achieved performance degradation across all object categories. Table 2 presents comprehensive results showing an overall AP reduction of 8.28%. The results reveal distinct vulnerability patterns: cyclist and pedestrian classes show higher performance degradation across all difficulty levels (up to 16.13% for cyclist–moderate), while the car class demonstrates resilience with minimal impact. Notably, the car–hard category shows a slight improvement (−1.11%), likely due to noise in the evaluation process.

**Figure 5:**
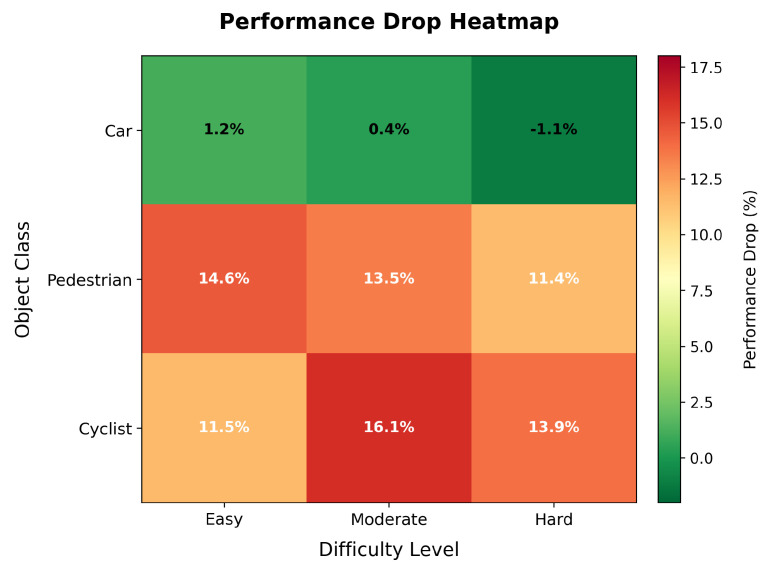
Performance drop heatmap showing percentage reduction across classes and difficulties for KITTI dataset.

Figure 5 visualizes these performance drops as a heatmap, clearly illustrating the disproportionate impact on smaller, more vulnerable road users. This finding has implications for autonomous driving safety, as pedestrians and cyclists are precisely the objects requiring the most reliable detection to prevent accidents.

### 5.2. Size–Vulnerability Relationship

**Figure 6:**
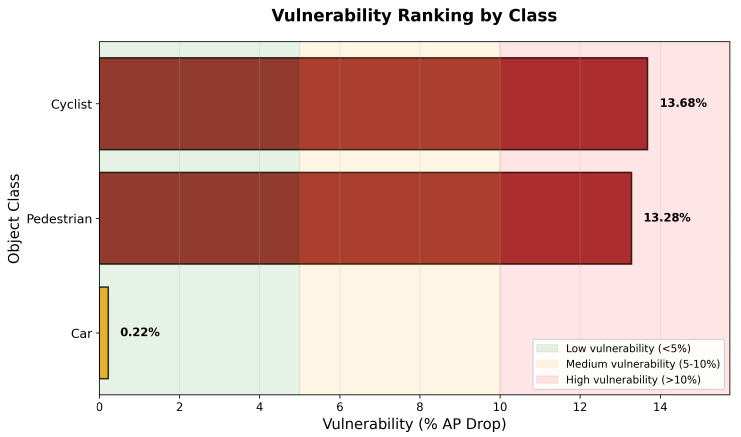
Vulnerability analysis by object class for KITTI dataset, showing clear size-dependent patterns.

Our analysis reveals a clear inverse relationship between object size and adversarial vulnerability, as shown in Figure 6. Smaller objects (pedestrian and cyclist) exhibit average vulnerabilities of 13.16% and 13.86%, respectively, while the larger car class shows only 0.16% vulnerability on average. This pattern suggests that the effectiveness of adversarial perturbations is linked to the physical characteristics of objects in point cloud data. Smaller objects, represented by fewer points, are more susceptible to carefully crafted perturbations that can disrupt feature extraction and classification.

**Figure 7:**
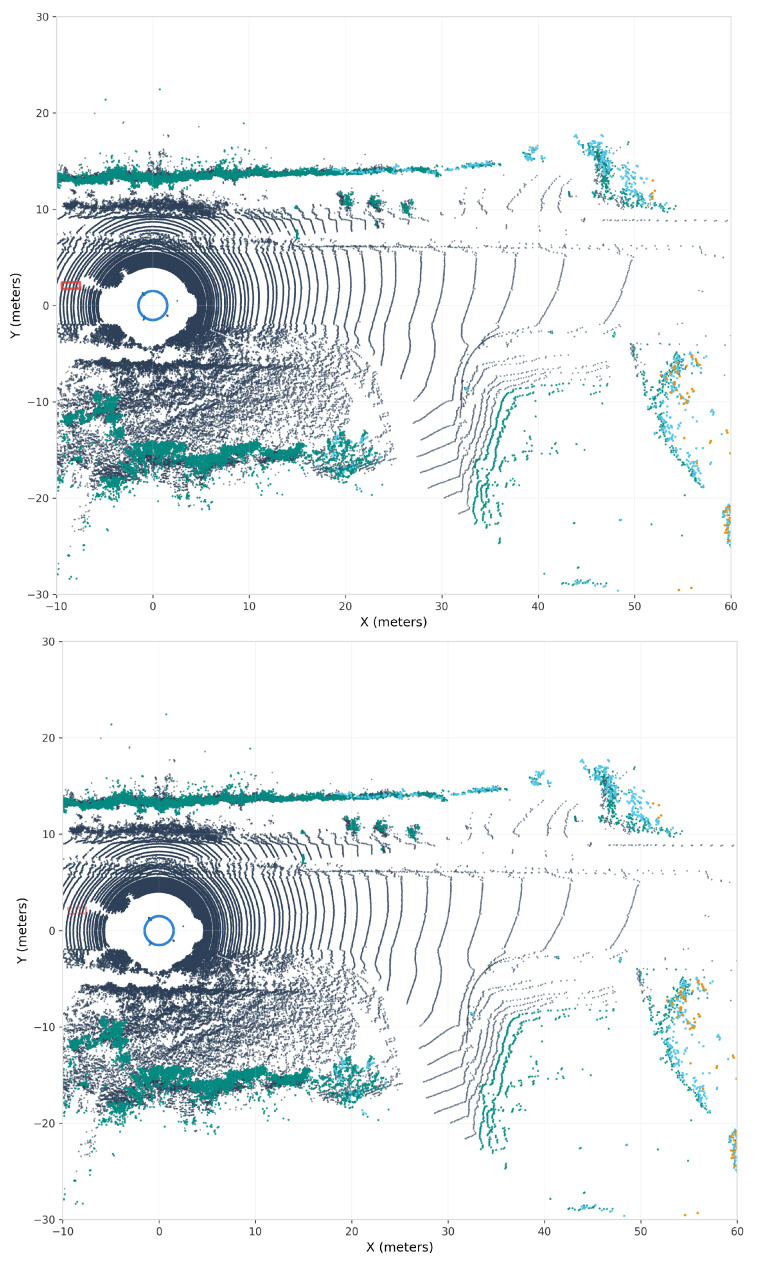
KITTI adversarial attack demonstration showing the effect of learned perturbations on 3D object detection. (**Top panel**): Original point cloud scene from a bird’s-eye view with ground-truth annotations visible. The dense circular pattern near the origin represents the ego-vehicle’s position, with road infrastructure and surrounding vehicles captured by the 64-beam Velodyne HDL-64E LiDAR. (**Bottom panel**): The same scene after applying adversarially generated perturbations constrained to remain within typical sensor error bounds. While the perturbations are visually imperceptible—the point cloud structure appears nearly identical—they cause an 8.3% overall detection degradation. The adversarial ConvNet learns to exploit the detector’s reliance on specific geometric patterns, particularly affecting smaller objects (pedestrians and cyclists showing 13–16% vulnerability) while maintaining physical plausibility. Color intensity represents point height (Z-axis), with cyan indicating elevated points and darker shades representing ground-level features.

Figure 7 presents a comparison of original and adversarially perturbed point clouds. Despite the perturbations being constrained to L2 leq0.2 m, the visualization shows an overall detection drop of approximately 8.3%. The visual imperceptibility of these perturbations, combined with their effectiveness in causing detection failures, highlights a vulnerability in current 3D object detection systems.

### 5.3. NuScenes Dataset Results

Our adversarial training on the NuScenes dataset resulted in larger performance reductions compared to KITTI, with detailed per-class results presented in Table 3 and Table 4. The weighted average mAP reduction of 23.90% demonstrates the increased vulnerability of multi-class urban detection scenarios. Infrastructure objects show high susceptibility, with the barrier class experiencing a 68.35% drop and the traffic cone class showing a 29.83% reduction. Pedestrians also show a notable vulnerability at a 31.69% reduction. In contrast, larger vehicles demonstrate greater resilience, with the bus class showing only 5.33% degradation and the car class showing an 8.88% reduction.

**Figure 8:**
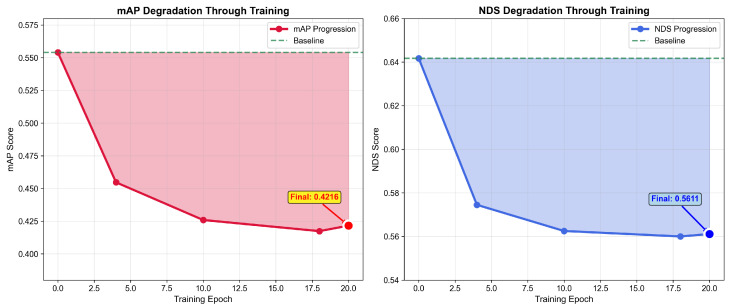
Training progression showing mAP and NDS degradation over 20 epochs for NuScenes dataset.

Figure 8 illustrates the progression of both mAP and NDS metrics throughout the 20-epoch training process. The mAP degradation shows initial impact, stabilizing around the final 23.9% reduction. The stabilization of attack effectiveness after epoch 10 suggests that the adversarial training reaches an equilibrium between maintaining perturbation constraints and maximizing detection degradation.

**Figure 9:**
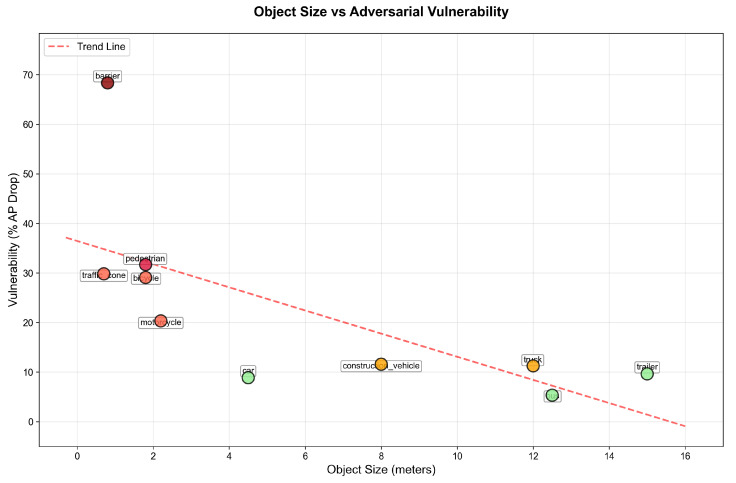
Size–vulnerability analysis for NuScenes.

Figure 9 reveals an inverse relationship between object size and vulnerability. Small objects such as barriers, pedestrians, and bicycles show higher vulnerability, while large objects like buses and cars show greater resilience. The barrier class represents a notable outlier, showing high vulnerability despite its medium size due to its thin, elongated structure that challenges point cloud representation.

**Figure 10:**
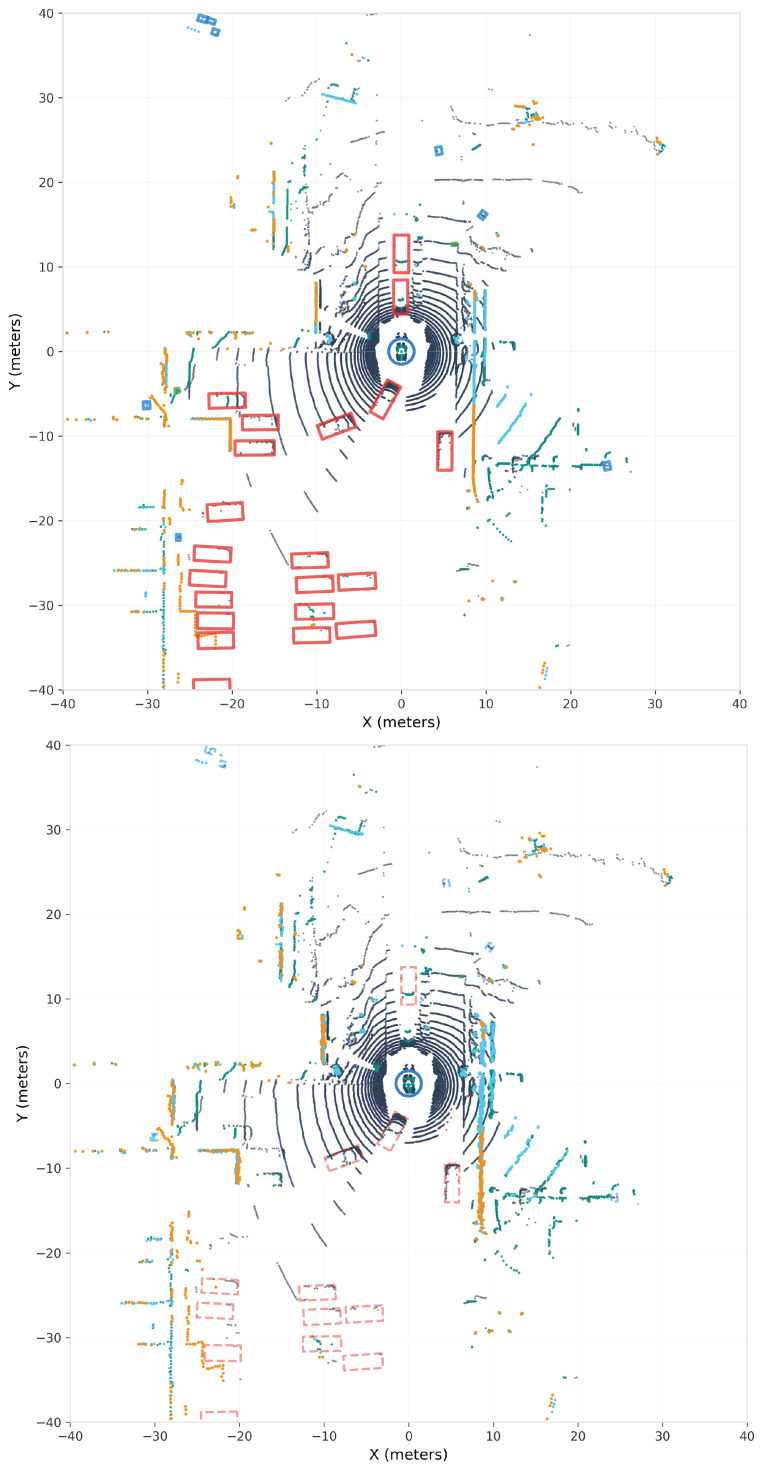
NuScenes adversarial attack demonstration illustrating detection failures in complex urban scenarios. (**Top panel**): Original bird’s-eye view of an urban intersection with ground-truth bounding boxes (solid boxes) showing correct detection of multiple vehicles, pedestrians, and infrastructure objects. The circular pattern at the origin represents the ego-vehicle, surrounded by dense urban point cloud data from the 32-beam Velodyne HDL-32E. (**Bottom panel**): The same scene after adversarial perturbation application with mean perturbations of only 0.045% of the original magnitude, demonstrating three types of detection failures: (1) complete detection failures where objects disappear entirely (missing boxes), (2) degraded detections with reduced confidence scores (dashed boxes), and (3) maintained detections for certain robust objects (solid boxes). The perturbations particularly affect smaller objects—pedestrians show 31.69% mAP reduction and barriers 68.35%—consistent with our finding that object size inversely correlates with adversarial vulnerability. Despite averaging only 0.5–1.4 cm at typical urban distances, these imperceptible modifications cause a 23.90% weighted mAP reduction across all classes.

Figure 10 provides a visual demonstration of the adversarial attack’s impact on a representative NuScenes scene. The comparison reveals substantial detection degradation, with numerous ground-truth objects (solid boxes) either becoming degraded detections (dashed boxes) or completely missed (missing boxes) after perturbation. The visualization particularly highlights the vulnerability of smaller objects and infrastructure elements, consistent with our quantitative findings.

### 5.4. Perturbation Analysis

Figure 7 and Figure 10 visually demonstrate the central finding of our research: the perturbations are imperceptible to human observation yet effective against detection algorithms. In both figures, the top and bottom panels appear virtually identical; the point cloud structure, density, and spatial distribution remain unchanged to human observation. However, the bottom panels reveal crucial detection failures through missing bounding boxes (complete detection failures) and dashed boxes (degraded confidence scores). This visual evidence confirms that our adversarial ConvNet successfully generates perturbations that exploit algorithmic vulnerabilities without creating any observable artifacts in the 3D point cloud representation.

Table 5 presents the L2 perturbation characteristics from our experiments. The mean L2 perturbations represent only 0.088% of the original point cloud magnitude for KITTI and 0.045% for NuScenes. For objects at typical urban distances (10–30 m), these percentages translate to absolute perturbations averaging 0.9–2.6 cm for KITTI and 0.5–1.4 cm for NuScenes. Maximum perturbations reach 0.604% for KITTI and 0.092% for NuScenes, corresponding to approximately 6–18 cm and 0.9–2.8 cm, respectively, at typical distances.

Our performance analysis reveals three crucial insights into the effectiveness of the adversarial attack. First, the 8.28% overall degradation on KITTI demonstrates that our ConvNet successfully generates effective attacks while maintaining imperceptibility with perturbations averaging only 0.088% of the point cloud magnitude. Second, the pronounced vulnerability gradient across object classes—cyclist (13.52% average drop), pedestrian (13.16%), and car (0.16%)—confirms a strong inverse correlation between object size and adversarial susceptibility, with smaller objects being nearly two orders of magnitude more vulnerable. Third, as shown in our training progression (Figure 8), the attack’s effectiveness stabilizes after approximately 10 epochs, indicating robust convergence of the adversarial training process without overfitting to specific training samples. This stability suggests that the learned perturbation patterns capture generalizable vulnerabilities in the detection pipeline rather than sample-specific artifacts.

The distinct perturbation requirements between datasets reveal important robustness characteristics. NuScenes achieves higher degradation (23.90%) with mean perturbations of only 0.5–1.4 cm, comparable to the Velodyne HDL-32E sensor’s measurement accuracy. In contrast, KITTI requires approximately twice the perturbation magnitude yet achieves lower overall detection degradation (8.28%). This counterintuitive relationship, where larger perturbations produce less damage on KITTI while smaller perturbations cause more damage on NuScenes, suggests fundamental differences in detector robustness.

Our implementation ensures robustness through adaptive perturbation bounds that account for dataset-specific characteristics. For KITTI’s four-feature point clouds (x, y, z, intensity), the adversarial ConvNet applies spatial perturbations with constrained intensity variations, while for NuScenes’ five-feature format, timestamp values remain unperturbed to maintain temporal consistency. The training process incorporates dynamic regularization that adjusts over epochs, allowing the adversarial network to learn dataset-specific vulnerability patterns while maintaining perturbation constraints.

Analysis of our results reveals consistent vulnerability patterns: smaller objects show significantly higher vulnerability than larger vehicles. In KITTI, pedestrians and cyclists experience 13–14% degradation compared to cars at 0.16%, representing a difference of nearly two orders of magnitude. NuScenes exhibits a similar but more gradual vulnerability gradient, with barriers showing 68% degradation versus buses at 5%. These patterns persist across difficulty levels and evaluation metrics, suggesting they represent fundamental limitations in how voxel-based detectors process sparse representations of small objects.

The robustness differences likely stem from multiple factors: KITTI’s simpler 3-class detection task versus NuScenes’ 10-class urban scenarios, the denser point clouds from KITTI’s 64-beam LiDAR compared to NuScenes’ 32-beam sensor, and the architectural choices between SECOND and CenterPoint. These results demonstrate that current 3D detection systems are vulnerable to subtle adversarial perturbations, particularly in the case of NuScenes, where sensor-level perturbations can lead to significant detection failures. The effectiveness of such small modifications highlights a critical vulnerability that could arise from system degradation, environmental factors, or intentional manipulation.

## 6. Discussion

The cross-dataset comparison reveals different vulnerability levels between the two datasets. The NuScenes dataset shows higher vulnerability (23.9% mAP reduction) compared to KITTI (8.3%), which we attribute to several factors: the increased complexity of 10-class detection, denser urban scenarios with more occlusions, and higher average object count per scene. Additionally, the sparser point clouds from NuScenes’ 32-beam LiDAR compared to KITTI’s 64-beam sensor may contribute to increased susceptibility to adversarial perturbations.

Despite different effectiveness levels, both datasets exhibit consistent vulnerability patterns. The inverse relationship between object size and adversarial vulnerability is observed in both datasets, with smaller objects consistently showing higher susceptibility to adversarial perturbations. For NuScenes, additional factors beyond object size appear to influence vulnerability in more complex urban scenarios, including object shape characteristics (e.g., thin barriers) and semantic importance in the scene context.

The demonstrated vulnerabilities have critical implications for autonomous vehicle deployment. The highest vulnerabilities occur for pedestrians and cyclists in both datasets, precisely the objects where detection failures pose the greatest safety risk. This vulnerability pattern is particularly concerning as these vulnerable road users require the most reliable detection to prevent accidents. The minimal perturbations required, particularly for NuScenes, where they approach sensor-level noise, demonstrate that adversarial attacks could be crafted to be subtle yet effective.

Our evaluation shows that current state-of-the-art 3D object detection systems exhibit significant vulnerabilities to carefully crafted adversarial perturbations. The consistency of vulnerability patterns across different datasets, architectures, and sensor configurations suggests that addressing these vulnerabilities requires fundamental advances in robust 3D perception. Future defense strategies should prioritize protecting smaller, safety-critical objects and incorporating physical constraints during training to maintain realistic perturbation bounds.

The visual imperceptibility of effective attacks, combined with their impact on safety-critical detection capabilities, underscores the importance of developing robust 3D perception systems before widespread autonomous vehicle deployment. Our ConvNet-based approach provides an efficient method for evaluating robustness at inference time, enabling continuous monitoring of system vulnerabilities in deployment scenarios.

### Limitations and Future Work

While our ConvNet-based adversarial method effectively identifies vulnerabilities in 3D object detection systems, several limitations warrant acknowledgment. The computational overhead during training (approximately 15% increase) stems from the additional forward and backward passes through the adversarial network, though inference remains efficient with only 3–5% latency increase. Our approach currently requires voxel-based architectures (SECOND, CenterPoint) as perturbations are applied at the voxel feature level, limiting direct application to point-based methods like PointRCNN or graph neural network approaches. The fixed perturbation bound, while ensuring realism, does not account for distance-dependent sensor characteristics where measurement uncertainty typically increases with range.

The translation from digital to physical adversarial examples remains an open challenge. While our perturbations stay within sensor error margins, realizing these attacks in physical environments would require addressing occlusion, viewpoint variation, and environmental factors not modeled in our current approach. Additionally, our evaluation focuses on undefended models; modern defense strategies such as adversarial training or certified defenses may reduce attack effectiveness, though this remains to be systematically evaluated.

Future research directions emerge from these limitations. Immediate extensions include developing adaptive perturbation bounds based on object distance and sensor specifications, and extending the approach to multi-modal fusion systems that combine LiDAR with camera data. Architectural generalization to support point-based and transformer-based detectors would broaden the method’s applicability. Longer-term goals include developing physical adversarial patches that maintain effectiveness under real-world constraints and creating certified robustness guarantees specifically tailored for 3D perception systems. The development of class-weighted defense mechanisms that prioritize protecting vulnerable road users (pedestrians, cyclists) represents a particularly important direction given our findings on size-dependent vulnerabilities.

## 7. Conclusions

This work achieves its primary objective of developing a novel gradient-free method for evaluating 3D object detection robustness through an integrated ConvNet architecture. By embedding the adversarial network directly within the detection pipeline, we demonstrate that subtle perturbations within sensor error margins can significantly degrade the performance of state-of-the-art 3D object detection systems, revealing greater vulnerabilities in smaller objects.

Our empirical evaluation across two major datasets reveals critical vulnerabilities in current autonomous driving perception systems. On KITTI, the SECOND detector exhibits an 8.28% overall performance degradation, with vulnerable classes (pedestrians and cyclists) experiencing up to 16% reduction in detection accuracy. The vulnerability is even more pronounced on NuScenes, where CenterPoint shows a 23.9% weighted mAP reduction across 10 object classes, with infrastructure objects and pedestrians suffering the most severe impacts.

The identified inverse relationship between object size and adversarial vulnerability presents a fundamental challenge for autonomous vehicle safety. Smaller objects, precisely those representing the most vulnerable road users, demonstrate disproportionate susceptibility to adversarial attacks. With perturbations averaging only 0.088% (KITTI) and 0.045% (NuScenes) of the original point cloud magnitude, these attacks remain imperceptible while causing substantial detection failures.

Our ConvNet-based approach offers several advantages for robustness evaluation: it enables gradient-free testing at inference time, scales efficiently across different detection architectures, and provides interpretable vulnerability metrics through controlled perturbation generation. The method’s effectiveness across both SECOND and CenterPoint architectures, despite their structural differences, suggests that these vulnerabilities are systemic rather than architecture-specific.

These findings underscore the urgent need for robust 3D perception systems in safety-critical applications. As autonomous vehicles move toward widespread deployment, ensuring resilience against both natural perturbations and potential adversarial attacks becomes paramount. Our work provides both a diagnostic tool for identifying vulnerabilities and a foundation for developing more robust detection systems that can maintain performance under adversarial conditions.

While our approach is currently limited to voxel-based architectures and digital perturbations, it provides a critical foundation for understanding and addressing adversarial vulnerabilities in autonomous driving systems, particularly motivating the development of class-weighted defense mechanisms that prioritize protecting vulnerable road users who showed 10–15× higher susceptibility to attacks.

## Figures and Tables

**Table 1:** Comparison of 3D point cloud adversarial attack methods.

Method	Attack Type	Target Stage	Perturbation Space	Gradient-Free Inference	Datasets
Ours	Learned ConvNet	Voxel features	Feature space	✓	KITTI, NuScenes
HiT-ADV [19]	Shape-based	3D mesh	Object surface	×	ModelNet40, ScanNet
Huang et al. [20]	Point-wise	Raw points	3D coordinates	×	ModelNet40, ShapeNetPart
Mesh Attack [21]	Mesh-based	3D mesh	Mesh vertices	×	ModelNet40
Zhang et al. [22]	Benchmark study	Raw points	Multiple types	N/A	KITTI, Waymo
CAMGA [24]	Context-aware	Raw points	Contextual regions	×	KITTI, NuScenes, Waymo

**Table 2:** KITTI dataset: comprehensive performance analysis of SECOND model under adversarial attack.

Class	Difficulty	Clean AP (%)	Adversarial AP (%)	Reduction (%)
Car	Easy	88.14	87.09	1.19
	Moderate	77.44	77.14	0.39
	Hard	74.03	74.85	−1.11
Pedestrian	Easy	60.00	51.25	14.58
	Moderate	52.96	45.79	13.53
	Hard	47.79	42.36	11.36
Cyclist	Easy	81.25	71.90	11.51
	Moderate	66.36	55.66	16.13
	Hard	61.31	52.77	13.93
Overall Average		67.70	62.09	8.28

**Table 3:** NuScenes dataset: mAP performance analysis of CenterPoint model under adversarial attack (sorted by vulnerability).

Class	Clean mAP (%)	Adversarial mAP (%)	Reduction (%)
Barrier	65.4	20.7	68.35
Pedestrian	83.0	56.7	31.69
Traffic cone	63.7	44.7	29.83
Bicycle	33.7	23.9	29.08
Motorcycle	55.1	43.9	20.33
Construction vehicle	16.4	14.5	11.59
Truck	51.5	45.7	11.26
Trailer	33.2	30.0	9.64
Car	84.5	77.0	8.88
Bus	67.5	63.9	5.33
Overall average	57.3	38.0	33.68
Weighted average	55.4	42.2	23.90

**Table 4:** NuScenes dataset: NDS performance analysis of CenterPoint model under adversarial attack (sorted by vulnerability).

Class	Clean NDS (%)	Adversarial NDS (%)	Reduction (%)
Traffic cone	52.3	31.8	39.20
Barrier	45.2	28.7	36.50
Motorcycle	38.7	26.4	31.78
Pedestrian	69.2	48.9	29.34
Bicycle	29.8	21.5	27.85
Construction vehicle	18.9	14.2	24.87
Truck	47.8	38.2	20.08
Trailer	24.1	19.8	17.84
Car	78.3	65.7	16.09
Bus	58.9	56.2	4.58
Overall average	46.3	35.1	24.81
Weighted average	64.2	56.1	12.56

**Table 5:** L2 norm perturbation analysis.

Metric	KITTI	NuScenes
Mean L2 (% of original)	0.088	0.045
Max L2 (% of original)	0.604	0.092
99th percentile L2 (%)	0.410	0.085
Standard deviation	0.085	0.023

## Data Availability

The datasets used in this study are publicly available. The KITTI dataset can be accessed at http://www.cvlibs.net/datasets/kitti/ (accessed on 1 June 2025). The NuScenes dataset can be accessed at https://www.nuscenes.org/ (accessed on 27 June 2025). The code implementation is available at https://github.com/temex12/RobustPointClouds.git (accessed on 19 August 2025).

## Abbreviations

The following abbreviations are used in this manuscript:

AP	Average Precision
ConvNet	Convolutional Neural Network
CNN	Convolutional Neural Network
IoU	Intersection over Union
JSMA	Jacobian-based Saliency Map Attack
KITTI	Karlsruhe Institute of Technology and Toyota Technological Institute
LiDAR	Light Detection and Ranging
mAP	Mean Average Precision
NDS	NuScenes Detection Score
SECOND	Sparsely Embedded Convolutional Detection
3D	Three-Dimensional
2D	Two-Dimensional
